# Improving modelling for epidemic response: a progress update from a community of UK infectious disease modellers

**DOI:** 10.1098/rsfs.2025.0007

**Published:** 2025-09-26

**Authors:** Katharine Sherratt, Anna C. Carnegie, Adam Kucharski, Anne Cori, Carl A. B. Pearson, Edward M. Hill, Elizabeth Fearon, Emily Nightingale, Julian Villabona-Arenas, Li Pi, Nicholas G. Davies, Sabine van Elsland, Sebastian Funk, Yang Liu, Sam Abbott

**Affiliations:** ^1^ Centre for Mathematical Modelling of Infectious Diseases, London School of Hygiene and Tropical Medicine, London, UK; ^2^ Social, Genetic and Developmental Psychiatry Centre, King's College London, London, UK; ^3^ MRC Centre for Global Infectious Disease Analysis, School of Public Health, Imperial College London, London, UK; ^4^ Atlantic Coast Center for Infectious Disease Dynamics and Analytics, University of North Carolina, Wilmington, NC, USA; ^5^ Civic Health Innovation Labs and Institute of Population Health, University of Liverpool, Liverpool, UK; ^6^ Institute for Global Health, University College London, London, UK; ^7^ Big Data Institute, University of Oxford, Oxford, UK; ^8^ NDM Centre for Global Health Research, Nuffield Department of Medicine, University of Oxford, Oxford, UK

**Keywords:** modelling, epidemic, outbreak response, pandemic preparedness, infrastructure, metascience

## Abstract

We reflect on the sustainability of modelling infectious disease outbreaks from the perspective of modelling as a field of practice. We formed a community of practice among UK infectious disease modellers who had contributed to the UK COVID-19 response. We previously used a participatory workshop approach to highlight issues in the infrastructure and incentives for outbreak modelling, and synthesized our experience into a set of 12 specific recommendations. Here, we track changes in the field of infectious disease modelling 1 year later, collecting the quantitative and qualitative views of change among 14 participants. We found participants continued to highlight a lack of ongoing, sufficient or appropriate action to develop outbreak modelling capacity in the UK, while positively noting collaborations among public health facing institutions. We emphasize the under-prioritization of funding for outbreak modelling outside of emergency response periods, and the continuation of unsustainable working practices. Correcting this is crucial to supporting evidence-based public health policy for outbreak preparedness and response.

## 1. Introduction

Infectious disease outbreaks can occur with little warning, requiring rapid action. This action can be supported by timely outbreak modelling. At the earliest stage, modelling may be a key decision support tool [1]. To provide insight into fast-paced outbreak dynamics, modellers must rapidly adapt their methods to each outbreak’s novel characteristics. This ‘trial by fire’ of modelling capacity can expose weaknesses across the structures and processes of modelling work [2]. Evaluating and addressing these limitations are fundamental to epidemic preparedness and response, and major outbreaks typically prompt a range of ‘lessons learned’ commentaries recommending improvements to the field of infectious disease modelling. As a community of UK modellers, we also contributed our perspective and call for change following COVID-19 [3].

However, translating evaluation into sufficient and appropriate action remains challenging. During an outbreak, allocating attention and resources to structural issues is hard to justify given the immediate need for crisis response. However, after an outbreak, the salience of these problems diminishes, and must compete with other scientific and professional priorities that were equally neglected during the crisis. The COVID-19 response and aftermath highlighted this pattern. We see that a variety of new collaborations and institutions now explicitly aim to improve policy-oriented outbreak modelling [4–8]. However, it is not clear how such initiatives map to existing challenges of the UK science–policy interface [9,10].

## 2. Tracking changes in the UK modelling landscape

We first came together as a community of 27 modellers in 2023 to reflect on our immediate experience of the UK COVID-19 response. We aimed to support more efficient, ethical and sustainable outbreak response modelling in future. We used a participatory workshop approach to represent experiences of outbreak response work across institutions and career stages. We synthesized this into a set of 12 specific recommendations [3]. In this piece, we aimed to track any corresponding changes in the modelling landscape.

**Figure 1.**
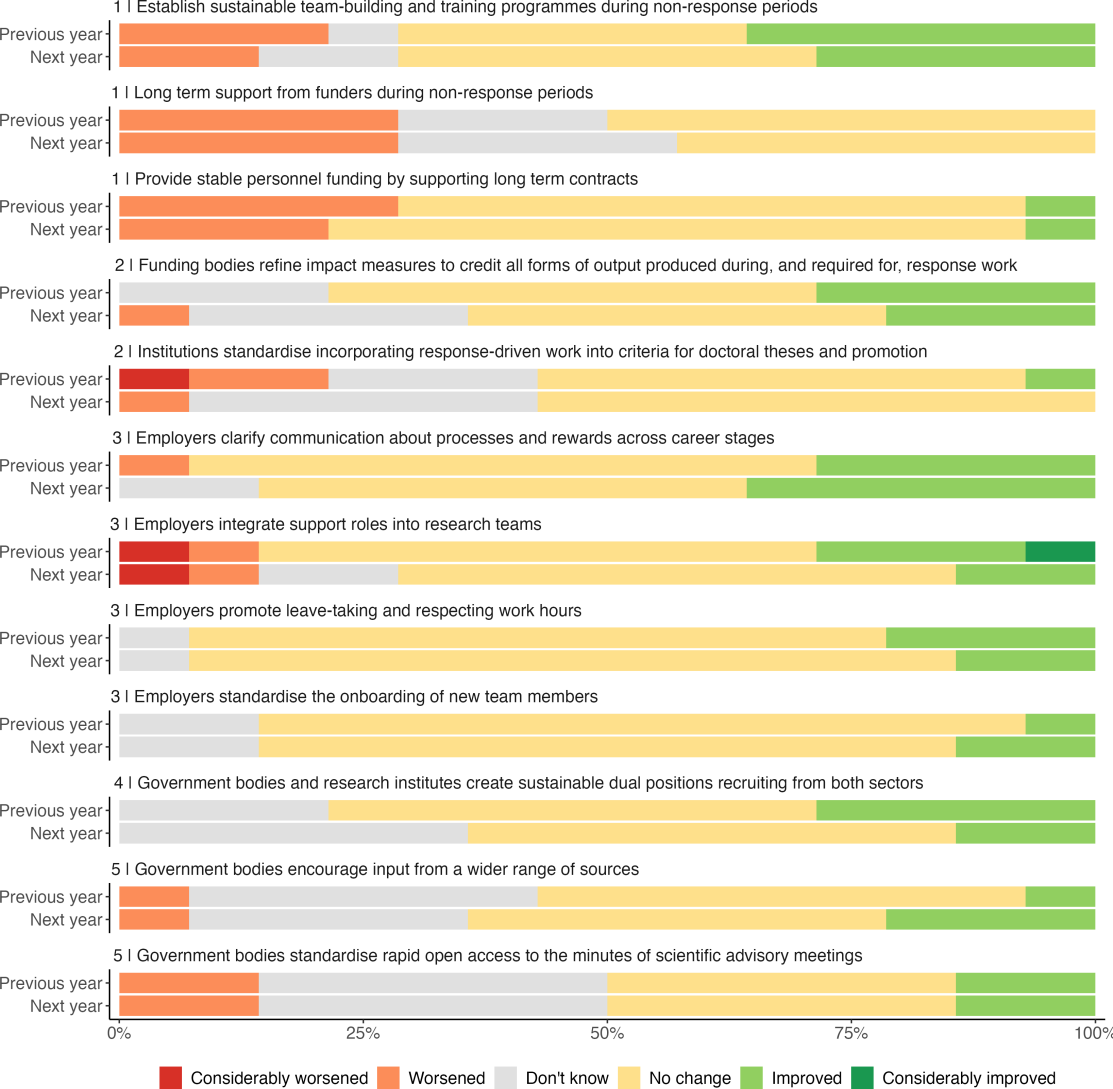
Views of recommended actions for outbreak response modelling as of April 2024 among 14 survey responders in the UK modelling community. Respondents were asked whether each situation had improved or worsened over the past year, and what their expectations were for the next year, across five priority areas with 12 recommended actions for improvement.

In March 2024, three organizers of the workshop (K.S., S.A. and Y.L.) re-contacted each author of the previous work. We used an online survey to ask participants whether the situation had improved or worsened over the past year, considering each of the 12 recommendations, and whether they expected the situation to improve or worsen over the next year. We used a 5-point ordinal scale (from ‘considerably worsened’ to ‘considerably improved’), with space for free text comment. Of 27 participants contacted from the 2023 workshop, 14 participants responded (figure 1). The results were collated and summarized by one author (K.S.) with review from two further authors (S.A. and Y.L.) and feedback from all participants, named as co-authors in this work. Anonymized data and code are publicly available [11].

Among participants, 13 worked in universities, with one in a public health agency, during the COVID-19 pandemic; only one had now left work in outbreak modelling. Each recommended action saw mixed views of positive and negative change. For all 12 recommended actions, participants most often reported ‘no change’ (accounting for 54% of all 366 responses) or ‘don’t know’ (19%). As in the previous work, we summarize participants’ comments into five thematic priorities.

### 2.1. Ensuring well-staffed, well-resourced, stable response teams with psychological support

Participants expressed the most negative views of change in sustainable support for response teams. Challenges focused on long-term funding and fixed-term contracts, reflecting structural issues across the UK academic sector (‘this all seems status quo*’*). Specifically, long-term support from funders during non-response periods was reported as either unchanged or worsened, with no expectation of future improvement (‘funders and the public have already lost interest in epidemic preparedness’). Several participants mentioned positive efforts to support staff training. However, this was also offset against staff turnover (e.g. ‘Training provided for lots of staff, but many staff employed on fixed-term contracts due to short term funding’).

### 2.2. Acknowledging and rewarding impactful response work across institutions, funders and research community

Some participants identified funding bodies adapting to give greater credit to response outputs (e.g. ‘UKRI has adopted a changed CV documentation for funding proposals’), with a positive impact on grant applications. In contrast, several participants had particularly negative experiences around acknowledgment of response work in criteria for theses and promotion (e.g. ‘Committees setting the criteria for doctoral theses and promotion have specifically chosen to exclude response work’), with low expectations for future improvements on these recommendations.

### 2.3. Implementing best practices for a sustainable work environment

Participants reported little change in the sustainability of work environments, against recommendations for clarifying career progression and integrating support roles, promoting leave-taking and standardizing team on-boarding (‘staff…are burnt out and over-worked…the fact that this has continued outside of the covid response context makes it even more insidious as it appears to have become the status quo’)*.* There was some optimism for future improvements in clearer communication about career progression. Participants had strong but mixed views on the progress of integrating support roles into research teams. Respondents noted both an increase in research software engineers, but also redundancies and a lack of acknowledgment of professional support staff (‘Support staff roles fail to be properly understood…or supported to develop’)*.*

### 2.4. Encouraging routine interaction between academia and public health agencies

While many participants indicated no change, several suggested some improvement in the presence of dual roles between government and research over both the past year and the future. Participants mentioned a strong recent interest in collaboration and an increase in collaborations between academia, public health agencies and the civil service (‘a lot of keenness for collaboration between academic modelers and UKHSA [the UK Health Security Agency]’; ‘I have seen efforts made to…promote inter-agency working… I am unsure how widespread or sustained they really are’).

### 2.5. Increasing transparency in the evidence pathway from scientists to decision makers

Participants more often reported ‘don’t know’ against changes in the recommended actions in this area. While some participants expressed cautious optimism, citing better access to data and the UK COVID-19 Inquiry [10] as examples of transparency (‘I think the COVID inquiry has put pressure on standardizing open access’), this was caveated with a lack of substantive change so far and several participants suggested a reversion to the status quo (‘I don't know of any mechanism in place yet to ensure this happens prospectively’; ‘I am not sure if any changes have formally been made…I am choosing to be optimistic’)*.*

## 3. What next for outbreak modelling?

This progress update highlights a lack of ongoing, sufficient or appropriate action to develop outbreak modelling capacity in the UK. We believe that this is partly a symptom of wider challenges across the UK university sector that exacerbate the poor fit between academic career structures and outbreak response modelling requirements. At the same time, we note similar issues, including high turnover in recent US initiatives, suggesting broad structural tensions across the field. Correcting this is crucial to supporting evidence-based public health policy for outbreak preparedness and response.

We are aware that we are a small, self-selected group from the original participants of the workshop within the modelling community. This means that our views may be impacted by selection bias, as it is likely that engagement over time is related to the strength of individuals’ views. We would be keen to see more structured and representative research that evaluates the infrastructure and impact of outbreak modelling work.

At the same time, our recommendations are consistent with other modelling evaluations, both pre- and post-COVID-19. Similar work calls for a stable ‘critical mass’ of modelling expertise, maintained at an institutional level independent of individual grant funding [12–15]. We emphasize the corresponding need for a sustainable research environment, considering and mitigating the differential impacts of response work on individuals [16–18]. In the specific context of response-mode work, we echo enduring calls for both tighter integration and greater transparency between scientific and policy operations [2,19].

In this progress update, we continue to see the under-prioritization of funding outside of emergency response periods. This is essential to create a base of trained personnel with the required skill set to effectively prepare for and respond to outbreaks. We also saw highly uncertain responses about what actions were being taken to change the field, possibly indicating a lack of progress in improving transparency, communication and integration. We emphasize these as priorities for further action. We further recommend that institutions aiming to improve outbreak response modelling should measure and report their structures and processes for supporting outbreak modelling, aligning with our well-supported set of recommendations for the field.

## Data Availability

All data and code are publicly available and archived at OSF [11].
